# Trends in research on novel antidepressant treatments

**DOI:** 10.3389/fphar.2025.1544795

**Published:** 2025-01-27

**Authors:** Agnieszka Zelek-Molik, Ewa Litwa

**Affiliations:** ^1^ Department of Brain Biochemistry, Maj Institute of Pharmacology, Polish Academy of Sciences, Krakow, Poland; ^2^ Department of Pharmacology, Maj Institute of Pharmacology, Polish Academy of Sciences, Krakow, Poland

**Keywords:** depression, animal models, RAAD, TRD, antidepressants, ketamine, psylocin

## Abstract

Mood disorders, such as major depressive disorder and bipolar disorder, are among the most common mental illnesses and a leading cause of disability worldwide. Key symptoms of these conditions include a depressed mood or anhedonia, sleep and psychomotor disturbances, changes in appetite or weight, and fatigue or loss of energy. Prolonged cognitive disturbances further impair the ability to think or concentrate and are often accompanied by persistent feelings of worthlessness or excessive guilt. Collectively, these symptoms underscore depression as a serious, long-term global health issue. In addition, clinical studies indicate a growing number of patients experiencing difficulties in responding to treatment, even in the long term. This phenomenon poses significant challenges for healthcare professionals, families, and patients alike. As a result, there is an urgent need for therapies that are both rapid-acting and safe. This review aims to summarize the prevailing trends in research on novel antidepressants, emphasizing their diversity and multi-directional mechanisms of action. The development of rapid-acting drugs is increasingly focused on achieving high efficacy, particularly for treatment-resistant depression. Such advances offer the potential for rapid therapeutic effects without the prolonged and often tedious administration of older generation antidepressants. Findings from studies using animal models of depression continue to play a crucial role in predicting and designing new therapeutic strategies. These models remain indispensable for understanding the physiological effects of newly developed compounds, thereby guiding the creation of innovative treatments.

## 1 Introduction

Major depressive disorder (MDD) is a common and growing global health concern, affecting over 264 million people, with an estimated lifetime risk of 15%–18% (Murray et al., 2012; Bromet et al., 2011). Unlike short-lived, everyday mood fluctuations, depression represents a severe and persistent health condition. Patients report many comorbidities that reduce quality of life and life expectancy (Berk et al., 2023).

According to the DSM-5 (Diagnostic and Statistical Manual of Mental Disorders, Fifth Edition), a diagnosis of a major depressive episode requires the presence of five or more symptoms for at least 2 weeks. Key symptoms include a depressed mood or anhedonia, sleep and psychomotor disturbances (agitation or retardation), changes in appetite or weight, and fatigue or loss of energy. Cognitive impairments, such as difficulties with concentration or decision-making, often accompany persistent feelings of worthlessness or excessive guilt.

The World Health Organization (WHO), in the 11th revision of the International Classification of Diseases and Related Health Problems (ICD-11), conceptualizes depression as a syndrome characterized by a clinically recognizable set of reported experiences (symptoms) and observed behaviors (signs) associated with distress and impaired functioning (Cuijpers et al., 2023; International Advisory Group for the Revision of ICD-10 Mental and Behavioural Disorders, 2011). At its most severe, depression can lead to suicide, which the WHO identifies as the second leading cause of death among individuals aged 15–29, accounting for approximately 800,000 deaths annually (Hawkins et al., 2018; Jensen et al., 2023; Murphy et al., 2021).

The discovery and widespread use of the first antidepressants revolutionized depression treatment. Grounded in the widely accepted monoamine theory of depression, first- and second-generation antidepressants, including serotonin and norepinephrine reuptake inhibitors, provided significant insights into mechanisms of action and treatment options. However, clinical trials have shown that these compounds are not equally effective in patients (Nunez et al., 2022; Salzman, 1996; Price et al., 1987). Furthermore, they often require weeks or months to achieve full effectiveness, underscoring the need for more effective and rapid-acting treatments for MDD (U.S. Food and Drug Administration (FDA) 2021; European Medicines Agency (EMA) 2013; Rush 2007; Mourilhe and Stokes, 1998).

In addition, one of the major challenges in treating depression is that many people who are prescribed antidepressants do not fully respond to pharmacotherapy. About 30% of patients treated for major depression do not respond satisfactorily to initial treatment. A significant number of patients have a poor prognosis at follow-up, with up to 20% still affected 2 years after onset (Fava et al., 2003). The STAR*D trial reported that one in three patients with unipolar depression did not achieve symptomatic remission after multiple antidepressant trials (Trivedi et al., 2006), and patients with bipolar depression did not benefit from antidepressant treatment combined with mood stabilizers (Bowden et al., 2012). These data indicate that a significant number of patients experience a phenomenon known as treatment-resistant depression (TRD) or refractory major depressive disorder (Berlim and Turecki, 2007; Tundo et al., 2023; Fu et al., 2024). Several authors have suggested distinguishing between difficult-to-treat and treatment-resistant depression on the basis of the longitudinal course of the illness. However, the common distinction indicates a clinical state resulting from a lack of response to treatment. As evidenced by the fact that several definitions have been proposed, the construct of TRD is very heterogeneous (Murphy et al., 2021).

## 2 Monoamine system

Current treatments for MDD are predominantly based on the monoaminergic system, as the monoamine hypothesis of depression suggests that a decrease in serotonin, norepinephrine, and dopamine levels in the central nervous system underlies the condition’s pathophysiology. The effectiveness of antidepressants supports this hypothesis, as drugs that increase the levels of these neurotransmitters in the brain have consistently been shown to reduce symptoms of depression.

Four main classes of monoamine-targeting antidepressants have been developed: first-generation antidepressants (monoamine oxidase inhibitors [MAOIs] and tricyclic antidepressants [TCAs]), second-generation antidepressants (selective serotonin reuptake inhibitors [SSRIs] and serotonin-norepinephrine reuptake inhibitors [SNRIs]), and atypical antidepressants. Among these, SSRIs are the most commonly used today, as they primarily address the hypoactivity of monoamine neurotransmitter systems (Sharp and Collins, 2023). Guidelines from the National Institute for Health and Care Excellence (Leichsenring et al., 2023) and the American Psychiatric Association (APA, 2019) recommend selective serotonin reuptake inhibitors (SSRIs), among other options, as first-line treatments for moderate to severe major depression. They are generally better tolerated and safer in overdose situations compared to TCAs and other antidepressants like noradrenergic and specific serotonin antidepressants or MAOIs (Olfson and Marcus, 2009; NHS Information Centre, 2011). However, an important limitation of all these treatments is their delayed onset of action, often taking 3 weeks or more to produce noticeable effects. During this time, especially in younger populations, SSRIs may exacerbate pre-existing anxiety or suicidality (Walter et al., 2022; Jack et al., 2023; Lagerberg et al., 2023).

Antidepressants acting via the monoaminergic system include partial agonists targeting serotonin receptors such as 5-HT1A, 5-HT1B, 5-HT2A, 5-HT2C, 5-HT4, and 5-HT7. Other mechanisms involve serotonin transporter inhibitors, 5-HT1A receptor “biased” agonists, triple monoamine reuptake inhibitors, and MAO-A inhibitors. Psychedelics, such as psilocin, also act within this system (Lochmann and Richardson, 2018; Weilburg, 2004; Papp et al., 2024). Psilocin, the most extensively studied psychedelic, primarily targets serotonin receptors but ultimately enhances glutamate release and sustained excitatory neurotransmission in pyramidal neurons. This promotes a prolonged state of enhanced neural plasticity in corticolimbic circuits, a key factor in its antidepressant effects.

The antidepressant effects of psilocin are thought to be mediated through activation of the 5-HT2A-mGlu2 receptor complex, which is also crucial for the hallucinogenic behaviors induced by 5-HT2A receptor agonists (López-Giménez and González-Maeso, 2018; Erritzoe et al., 2024). Activation of 5-HT2A receptors promotes glutamate release in pyramidal neurons of the prefrontal cortex, leading to both antidepressant and anxiolytic effects. Research on mitigating the adverse acute effects of psilocybin suggests these effects are related to the occupancy of 5-HT2A receptors (Madsen et al., 2019; Lagerberg et al., 2023). Interestingly, chronic administration of SSRIs or SNRIs has been shown to lead to downregulation of 5-HT2A receptors in both animal and human studies (Kumar et al., 2019; Klimek et al., 1994; Meyer et al., 2001).

## 3 Glutamatergic system

Among newer, faster-acting pharmacological treatment strategies, those targeting the glutamatergic and GABAergic systems have shown considerable promise for the treatment of MDD and TRD. A particularly notable example is (R,S)-ketamine, whose rapid improvement in depressive symptoms after administration (Swainson et al., 2019; Berman et al., 2000; Barbara et al., 2024) has significantly renewed interest in compounds that modulate glutamatergic receptors, sparking a wave of research and drug development in this area. (R,S)-Ketamine acts as an NMDA receptor antagonist. Its S (+) isomer, esketamine, has been approved in the European Union, United Kingdom, and United States for adults with TRD and for the rapid reduction of depressive symptoms in psychiatric emergencies related to MDD. Ketamine’s effects, while mechanistically distinct from those of psilocin, share similarities in their activation of intracellular signaling pathways. These pathways produce long-lasting changes in synaptic function and morphology (neuroplasticity), proposed as a common mechanism underlying the therapeutic effects of ketamine and classical psychedelics (Aleksandrova and Phillips, 2021). While newly developed neuroplasticity-based strategies do not directly target glutamate modulation, they focus on activating the intracellular mammalian target of rapamycin complex 1 (mTORC1) signaling cascade. This pathway links glutamate modulation to the induction of neuroplastic changes.

Specific enantiomers, racemic mixtures or metabolites of ketamine have different affinities for NMDAR and have shown antidepressant activity in preclinical and clinical studies. Metabolites of ketamine such as S-norketamine, 2S,6S-hydroxynorketamine, 2S,6R-hydroxynorketamine and 2R,6S-hydroxynorketamine have shown antidepressant-like effects in animal models (Zanos et al., 2016; Yokoyama et al., 2020). Differences between (R)-ketamine and (S)-ketamine have been observed at behavioural and biochemical levels. Some preclinical studies also show that (R)-ketamine has stronger and longer-lasting effects and is safer than ketamine or (S)-ketamine. That suggest that (R)-ketamine could be a promising molecule and, like some metabolites, should be tested in clinical trials (Pochwat et al., 2022).

Among the many compounds under investigation, NMDA and metabotropic glutamate receptor antagonists and negative allosteric modulators appear particularly promising due to their ability to inhibit glutamatergic neurotransmission. These include broad-spectrum glutamatergic modulators, like some of the non-NMDA receptor agonists such as glycine site NMDAR partial agonists, NR2B antagonists, non-selective low-trapping NMDAR channel blockers or non-competitive NMDAR antagonists are active in models of depression (Machado-Vieira et al., 2017). NMDAR antagonists alone or as an adjunct to imipramine/SSRIs have produced antidepressant effects in animal models of depression (Poleszak et al., 2011).

Regarding metabotropic glutamate receptors, all three groups show antidepressant effects, especially under experimental conditions. For group I mGlu receptors, promising ligands include mGlu1 receptor antagonists, mGlu5 and partial mGlu5 receptor negative allosteric modulators, but also mGlu5 receptor inverse agonists. For group II mGlu receptors, antidepressant-like activity has been demonstrated by mGlu2/3 receptor agonists, mGlu2/3 receptor antagonists, and mGlu2/3 and selective mGlu2 and mGlu3 receptor negative allosteric modulators. To a lesser extent, group III mGlu receptors are being investigated. Group III mGlu receptor agonists, mGlu7 receptor negative allosteric modulators, mGlu7 receptor allosteric agonists and mGlu8 receptor agonists (Dogra and Conn, 2021). As shown, metabotropic glutamate receptor ligands excert antidepressant potential and, as some suggest, could be used as adjuncts to reduce the side effects of rapid-acting antidepressants (discussed in Pochwat et al., 2022).

Some experimental compounds exhibit intriguing multimodal mechanisms of action, including NMDA receptor antagonism combined with α-1 adrenergic receptor agonism. Additionally, a positive allosteric GABAA modulator is currently in phase III clinical trials (Clayton et al., 2023). It should be emphasised that a large number of NMDAR antagonists are effective in preclinical models of depression, but have been unsuccessful in clinical trials (Kishimoto et al., 2016). However novel glutamatergic modulators for the treatment of mood disorders encompass a diverse range of approaches. These advances are yielding new insights and opportunities for the development of rapid-acting treatments for MDD (for reviews, see Henter et al., 2021; Serretti, 2024).

## 4 Treatment-resistant depression

Treatment-resistant depression is a significant clinical challenge. While some studies report that over 35% of patients with depression are resistant to antidepressant treatment (Nemeroff, 2007; Thase, 2011; Thomas et al., 2013), more recent data highlight an even greater burden. According to Heerlein et al. (2021); Heerlein et al. (2022), approximately 69% of patients with difficult-to-treat depression in Europe did not respond to treatment within 1 year. Despite low remission rates, 60% of these patients remained on unchanged treatment regimens for extended periods. Some studies reported that in some populations even one-fourth of all depressive patients are affected by TRD (Gałecki et al., 2022). Mortality, particularly due to suicide and accidents, as well as comorbid conditions, is significantly higher in TRD patients (Döme et al., 2021; Reutfors et al., 2018; DiBernardo et al., 2018). Non-lethal self-harm is also more frequent in TRD patients compared to those without this condition (Parker and Graham, 2015). Furthermore, TRD is associated with worse disease outcomes and elevated rates of general and psychiatric comorbidities relative to other patients treated for depression.

Among recently introduced TRD treatments, esketamine has a special place. Recent studies confirm the efficacy, safety and safety and tolerability of esketamine in adults with TRD. In patients with comorbidities in the REAL-ESK study reported the later response, as well as the non-inferiority in effectiveness present novel and interesting findings (Martinotti et al., 2022). It should be noted that esketamine is only approved for TRD and emergency suicidality. It represents an important novelty in the pharmacological treatment of patients with depression, but its specific mechanism of action may be associated with craving behaviour and additional addictive potential (Leichsenring et al., 2023). A narrative review of the available literature on the most common clinical “misconceptions” and “stereotypes” about esketamine has recently been presented by Di Vincenzo et al. (2024).

Despite ongoing research, there is no universally effective treatment strategy for TRD. Current guidelines recommend several approaches, including dose optimization, switching antidepressants, or augmenting therapy with other pharmacological agents (Gabriel et al., 2023; Davies et al., 2019). The high heterogeneity in the treatment of patients with TRD and the urgent need for standardised strategies and treatments specifically approved for TRD are highlighted. In addition to esketamine, some new studies describe the benefits of adding lithium or antipsychotics to adjuvant therapies for patients with TRD (Maina et al., 2023). Strong recommendations also exist for disorder-specific psychological therapies, such as cognitive analytic therapy, cognitive behavioral therapy (including internet-based CBT), and interpersonal therapy for depression (Ijaz et al., 2018). However, long-term treatment often proves discouraging for both patients and their families, emphasizing the urgent need for alternative therapeutic approaches.

## 5 Optimizing treatment strategies for TRD

The latest guidelines advocate for first addressing pseudo-resistance by ensuring adherence to treatment and optimizing antidepressant dose and duration. For combination therapies, the pairing of monoamine reuptake inhibitors with presynaptic α2-adrenoceptor antagonists has shown promise in improving efficacy in TRD patients. Additionally, targeting the opioid system with µ-receptor agonists, mixed (µ/ĸ) receptor agonists, or ĸ-receptor antagonists may provide novel avenues for treatment (Borbely et al., 2021). Agonism of the 5-HT1A receptor is another promising strategy for achieving rapid and sustained therapeutic effects in TRD (Smith et al., 2023; Papp et al., 2023).

Among augmentation strategies, second-generation antipsychotics and lithium remain the most evidence-supported options for managing TRD. Novel therapies, particularly those targeting the glutamatergic system, have garnered significant attention. Intravenous ketamine and intranasal S-ketamine are at the forefront of these developments. Since intranasal S-ketamine was approved by the U.S. FDA and the EMA in 2019, it has been introduced as a novel adjunctive therapy for TRD with specific indications and under laboratory control. These therapies offer hope for addressing the limitations of traditional treatment approaches. We identified three primary clinical approaches to optimize treatment for TRD: neurostimulation therapy, metabolic modulation, and ion level modulation.

### 5.1 Neuromodulations/neurostimulations treatments

Neuromodulation is a growing area of research interest based on clinical and preclinical reports (Hamani and Nóbrega, 2010; Figee et al., 2022; Papp et al., 2018). Non-invasive techniques include electroconvulsive therapy (ECT), magnetic seizure therapy (MST), transcranial direct current stimulation (tDCS), and repetitive transcranial magnetic stimulation (rTMS). Invasive procedures include neurosurgical vagus nerve stimulation (VNS) and deep brain stimulation (DBS), in which electrodes are implanted in discrete brain targets under MRI guidance. For affective disorders, as recommended by the Canadian Agency for Drugs and Technologies in Health, 2014, DBS for MDD is still under investigation. Acute and maintenance efficacy, as well as safety and tolerability, were classified at level 3 in 2016 (Milev et al., 2016). This classification means that recommendations are based primarily on epidemiological or risk factor studies, observational studies, randomized controlled trials with small samples, non-randomized controlled prospective studies or case series, or high-quality retrospective studies.

### 5.2 Metabolic modulation

The unadaptable stress response with the prolonged energy mobilization promotes dysglycaemia and insulin resistance that in turn alter mitochondrial structure and function generating oxidative stress, and inflammation leading to cellular damage (Picard et al., 2014). This mechanism likely underlies distinguished recently metabolic subtype of MDD (Krupa et al., 2023) and justifies the frequent co-occurrence of type 2 diabetes (TMD2) and depression (Guerrero Fernández de Alba et al., 2020). Insulin resistance has been considered as a factor responsible for more severe depressive symptoms and as a predictor of lack of response to antidepressant drugs. It should be noted that both in the clinics and preclinical studies the insulin-sensitizing interventions improved the effectiveness of antidepressive treatments. The antidepressive mechanism of insulin applied in combined MDD therapy seems to be related to the direct modulatory tone of insulin into serotoninergic and dopaminergic neurotransmission, which is known to be disrupted in patients with insulin resistance (see Krupa et al., 2023).

Cellular metabolic overactivity during exposure to stressor(s) augments the cellular (mainly mitochondrial) production and accumulation of oxygen reactive species (ROS) which in physiological state are neutralized by antioxidant defensive system primarily consisting of superoxide dismutase (SOD), catalase (CAT), glutathione peroxidase (GPX), glutathione reductase (GR), and glutathione S-transferase (GST) enzymes. Oxidative stress, defined as an imbalance between production and accumulation of ROS in cells and tissues is considered as an important factor implicated in the pathomechanism of MDD (Bell et al., 2024; Lievanos-Ruiz and Fenton-Navarro, 2024; Correia et al., 2023). Recent data, performed by proton magnetic resonance spectroscopy methodology revealed reduced levels of GSH in the occipital region of the cortex of patients with MDD (Bell et al., 2024). Although the precise pattern of depression evoked changes in the level and/or function of the antioxidant defensive system is not clear, strategies reducing the oxidative stress components seem to be effective in the reduction of depressive symptoms in the clinics (Bajpai, 2014; Zhang et al., 2022) and in the preclinical studies (see Correia et al., 2023).

### 5.3 Ion levels modulation

All biochemical processes necessary for the proper intracellular signal transduction, protein synthesis, activity, and other metabolic pathways require homeostasis of trace elements including zinc (Zn2+), magnesium (Mg2+), calcium (Ca2+), copper (Cu2+), iron (Fe2+), and manganese (Mn2+) ions (Meng et al., 2024). The monitoring of trace elements may serve as a diagnostic panel and provide information about the effectiveness of antidepressive therapy applied to patients. Clinical observations regarding the serum levels of trace elements in depression patients pointed out decreased Zn2+, and Mg2+ serum levels as potential markers of depression and evidenced the beneficial effects of Zn2+, and Mg2+ supplementation in the treatment or prevention of depression (Szewczyk et al., 2018). Moreover, in the case of TRD, Zn2+ supplementation was shown to augment the efficacy of antidepressant pharmacotherapy (Siwek et al., 2010; Styczeń et al., 2016). Multiple preclinical studies supported clinical observation regarding the role of Zn2+, and Mg2+ deficiency in the expression of depressive-like behaviours (Szewczyk et al., 2018). Regarding the potential antidepressive mechanism of Zn2+, its role in direct activation of metabotropic GPR39 receptor is postulated, and modulation of GPR39 downstream CREB/BDNF/TrkB signalling and related neuroplasticity (Meng et al., 2024). In turn, antidepressive properties of Mg2+ seem to be related mainly to the blockade of the NMDA receptors overactivated in the forebrain in the presence of physical or anticipated stressors (Zelek-Molik et al., 2025). Additionally, Mg2+'s role in the regulation of the HPA axis, inflammation and oxidative stress, regulation of the glutamatergic, 5HT, dopamine and norepinephrine signalling and the sleep-wake cycle has been recognized. Available data suggest that neither depression nor antidepressant treatment affects the Fe2+ and Mn2+ levels. However, the concentration of Ca2+ and Cu2+ in the blood and brain of MDD patients was shown to be upregulated. Moreover, a positive correlation between the patient’s severity of depression and Ca2+ and Cu2+ levels was detected (Meng et al., 2024). Typically, the antidepressive treatment with SSRIs was shown to alleviate the augmented level of Cu2+, however, treatment with lithium additionally increased Ca2+ concentration and may lead to hypercalcemia, recognized as a harmful side effect after lithium treatment.

## 6 Trends in the search for biomarkers to discover more efficient antidepressants

To assess the latest approaches in studying molecular targets and testing antidepressive strategies in preclinical models of depression, we performed a literature search using the PubMed database with keywords such as “depressive-like behavior” and “pharmacotherapy”. This search yielded 191 review articles published within the last 5 years up to September 2024. After excluding irrelevant publications, non-English articles, and those restricted by open-access limitations, 64 scientific articles were selected, each describing currently tested targets and potential pharmacotherapies for depression.

Among the selected articles, a significant portion (43 articles) focused on the modulation of neuroinflammation, gut-brain axis activity, and BDNF related neuroplasticity processes in the forebrain. Additionally, ten articles explored the therapeutic potential of bioactive plant compounds, reflecting the growing interest in leveraging the therapeutic properties of plants in medicine.

Below, we summarize the most relevant findings from these reviews and provide references to the literature where readers can find detailed analyses and links to original studies.

### 6.1 Neuroinflammation

Impaired inflammatory responses have been identified as a significant factor in the pathomechanism of depression (e.g., Wang et al., 2024; Bielawski et al., 2023; Belzeaux et al., 2012). As mentioned above, depression-related neuroinflammation is connected to the excessive metabolic processes and oxidative stress occurrence (e.g., Correia et al., 2023). Treatment-resistant depression (TRD) and depressive-like behavior are associated with glial dysregulation, particularly in the frontal cortex and hippocampus (Sanadgol et al., 2023; Fang et al., 2023; Bansal et al., 2024; Furuyashiki et al., 2019). This dysregulation manifests as elevated levels of proinflammatory cytokines (Il1β, Il6, TNFα) in the blood and brain and overactivation of immune receptors (TNFR1 and TLR-4). These changes lead to the upregulation of the NF-κB pathway, heightened inflammatory responses, and depressive-like symptoms (Sanadgol et al., 2023; Sharma et al., 2024).

Additionally, the synthesis of proinflammatory lipid mediators such as prostaglandins and cysteinyl leukotrienes, linked to abnormal arachidonic acid turnover in the brain, has been observed in depressive-like behavior (Furuyashiki et al., 2019). Available antidepressants are largely ineffective in reducing neuroinflammation in TRD patients (Belzeaux et al., 2012) and in preclinical models (Duda et al., 2017; 2019), emphasizing the need to identify new therapeutic targets for depression-induced neuroinflammation.

One promising approach is targeting the NLRP3 inflammasome and its P2X7 receptor, which regulate the release of proinflammatory cytokines and are under investigation as potential antidepressive pharmacotherapy targets (Mokhtari and Uludag, 2023; Roy et al., 2023; Qi et al., 2023). Animal models have also shown that depressive-like behavior is accompanied by increased activity of fatty acid amide hydrolase (FAAH), an enzyme in the endocannabinoid system. FAAH inhibitors have demonstrated therapeutic potential without the side effects associated with direct CB1 agonists (Rafiei and Kolla, 2021). Furthermore, studies have revealed that increasing omega-3 fatty acid intake or its derivatives, such as resolvins, can attenuate neuroinflammation-related depression (Furuyashiki et al., 2019).

### 6.2 Gut-brain-axis

The gut microbiota is a key site of neurotransmitter synthesis, such as serotonin (5-HT), norepinephrine (NE), and dopamine (DA), which are impaired in depression (Bhatt et al., 2022). It is widely accepted that depression and depressive behaviours are associated with gut dysbiosis induced by excessive activation of the HPA axis and increased levels of cortisol/corticosterone in the blood. This results in increased intestinal permeability and increased translocation of gut bacteria and their metabolites (including short-chain fatty acids, neurotransmitters, and cytokines) into the circulation (a process also known as “leaky gut”) (Halvorson et al., 2024). A leaky gut causes increased inflammation, changes in gut bacteria (including reduced levels of Bifidobacterium and *Lactobacillus*), and accompanying symptoms of depression, which can be alleviated by taking probiotics and prebiotics (Sharma et al., 2024; Sjöstedt et al., 2021). Depression often accompanies irritable bowel syndrome (IBS), which is directly related to the occurrence of gut dysbiosis, effectively treated with SSRIs. The underlying mechanism of the therapeutic interaction between the microbiota and psychotic treatment is not clear, however. In contrast to the aforementioned therapeutic effects observed in SSRI-treated IBS, preclinical evidence shows that the metabolic disturbances observed with olanzapine are due to the drug-microbiota interaction, as the use of antibiotics that eliminate gut bacteria reversed the adverse side effects of olanzapine (Sjöstedt et al., 2021). Antidepressant therapy can also affect the composition of the microbiota. For example, MAO-A inhibitors (iproniazid) or SSRIs (sertraline, fluoxetine, paroxetine) have been shown to act like antibiotics, leading to microbial dysbiosis, which is associated with weight gain reported as a side effect after long-term SSRI use. In turn, vortioxetine, a 5-HT receptor modulator and SERT inhibitor (Sanchez et al., 2015) seems to exert its antidepressant effects by regulating the gut microbiota in patients with MDD (Ye et al., 2021). Understanding the precise mechanism linking dysbiosis with depression and dysbiosis induced by some antidepressants seems important for the development of improved antidepressants.

Currently, it is evidenced that gut microbiome communicates with the brain through the gut-brain axis, regulating stress responses, inflammation, and emotions (Guerrero-Hreins et al., 2021; Pinna, 2023). Glucagon-like peptide 1 (GLP-1), released from the colon, reaches the brain via the vagal afferent pathway and activates GLP-1 receptors (GLP-1R), which are widely expressed in limbic structures. This pathway is implicated in regulating feeding and emotional behaviors.

Clinical observations suggest that pharmacotherapy with GLP-1R agonists may induce demotivation and anhedonia with prolonged use (Dumiaty et al., 2024; Detka and Głombik, 2021). Preclinical studies have recently highlighted reelin, an extracellular matrix protein expressed in the brain and intestines, as a promising antidepressive compound (Halvorson et al., 2024). In the brain, reelin supports synaptic plasticity, while in the intestines, it interacts with receptors such as VLDLR, ApoER2, EphB2, and α3β1-integrins. Similarly to ketamine, reelin was shown to activate mTORC1, without NMDA receptors antagonism, suggesting that rescuing reelin level may offer rapid antidepressant effects avoiding the side (dissociative) effects related to therapy with ketamine (Johnston et al., 2023).

Epigenetic modifications of the gut microbiome and its metabolites, induced by diet, prebiotics, probiotics, specific antibiotics, fecal microbiota transplantation, or antidepressants, may have beneficial antidepressive effects (Nohesara et al., 2023). For example, augmentation of allopregnanolone (AP) signaling through PPAR-α (peroxisome proliferator-activated receptor α) in the colon has been shown to reduce gut dysbiosis and depressive symptoms (Pinna, 2023). Similarly, gastrointestinal peptides like ghrelin have emerged as potential targets for modulating dysbiosis-related depressive-like behavior (Gajewska et al., 2023).

### 6.3 Neuroplasticity

Dysregulation of neural plasticity is a well-established factor implicated in the pathophysiology of depression and is characterized by hypoactivation, synaptic loss and weakening, reduced connectivity, and decreased levels of neurotrophic factors (reviewed in Matar et al., 2024). Brain-derived neurotrophic factor (BDNF) is considered a critical mediator between antidepressant activity and the neuroplastic changes responsible for therapeutic effects. Impaired BDNF-TrkB signalling and its regulatory influence on NMDA and AMPA receptors in the forebrain leads to structural and functional changes in neurons and the development of psychiatric diseases, including depression (McGinty et al., 2015; Sun et al., 2014; Sharma et al., 2024; Zelek-Molik et al., 2021). Glutamatergic modulation of AMPA and NMDA receptors on cortical pyramidal neurons leads to upregulation of BDNF expression. Through this mechanism, glutamate release and AMPA receptor activation enhance BDNF binding to TrkB. This activates mTOR and ERK signalling, intensifying the expression of neuroplasticity-related genes, protein synthesis of synaptic components, and mechanisms that result in rapid and long-lasting local synaptogenesis (Pochwat et al., 2022).

Antidepressants enhance neuroplasticity through several pathways. By modulating the TrkB receptor and increasing the excitability of pyramidal neurons, these treatments amplify excitatory transmission. Receptor activity of antidepressants influences mTORC1 through serotonin transporters (as seen in classical antidepressants), NMDA, mGluR2, or AMPA receptors (in rapid-acting antidepressants like ketamine and its metabolites), 5HT2A/5HT1A receptors (as in psychedelics such as psilocin or lysergic acid diethylamide), or even through heterodimer formation, which potentiates neuroplasticity effects between receptor types (Brunello et al., 2024; Ilchibaeva et al., 2022). Further, downstream effectors including mTORC1, ERK, CREB, and elongation factor 2 (EF2) mediate intracellular effects of antidepressants by inducing plasticity-related genes and BDNF expression. These neuroplasticity-driven processes significantly reduce depressive symptoms by promoting synaptic remodelling and enhanced connectivity (Page et al., 2024; Moliner et al., 2023; Casarotto et al., 2021; Saarelainen et al., 2003). Although BDNF does not cross the blood-brain barrier (BBB), its levels in the brain have been shown to increase through physical exercise. This effect is mediated by the myokine irisin, a protein upregulated in muscle during exercise, which is cleaved from its precursor (FNDC5), secreted into circulation, and capable of crossing the BBB to stimulate BDNF synthesis in the hippocampus. This process supports neuroplasticity and related therapeutic outcomes (Colucci-D’Amato et al., 2020; Liu et al., 2024).

BDNF exerts its effects not only through high-affinity binding to TrkB receptors but also through low-affinity interactions with the neurotrophin receptor p75NTR and its co-receptor sortilin. These interactions activate a wide range of intracellular signalling pathways involved in cytoskeletal remodelling and plasticity. Recently identified downstream effectors of BDNF signalling that may serve as potential targets for new antidepressant therapies include non-receptor tyrosine kinases such as SFK/JAK/FAK (Wang et al., 2022) and FYN (Zelek-Molik et al., 2025); lysophosphatidic acid (LPA) and its receptors (Li and Li, 2024); the small GTPase RhoA (Rafa-Zabłocka et al., 2021); and adhesion G protein-coupled receptor GPR65 (Qi and Guan, 2024).

### 6.4 Pleiotropic antidepressive activity of plant compounds

Recent studies have highlighted the antidepressant potential of various phytochemicals, including gintonin (Kim H-J et al., 2017), ferulic acid (Dong and Zhao, 2023), ginsenoside Rg1 (Yang et al., 2022), flavonols (Jazvinšćak Jembrek et al., 2023), flavonoids (German-Ponciano et al., 2022; Ko et al., 2020), mitragynine (Johnson et al., 2020), and terpenoids such as linalool, linalyl acetate, geraniol, citronellol, and limonene (Muller et al., 2021; Agatonovic-Kustrin et al., 2020). While the precise mechanisms of antidepressant action and their clinical usefulness require further research, it has been observed that their effects often combine anti-inflammatory properties, improvements in brain-gut axis function, and positive effects on neuronal plasticity.

The anti-inflammatory effects of plant compounds have been linked to downregulation of the proinflammatory MAPK and NF-κB pathways (Wang et al., 2024; Dong and Zhao, 2023), upregulation of the heme oxygenase (HO) system via nuclear transcription factor erythroid 2-related factor 2 (Nrf2) (Wang et al., 2024), inhibition of MAO-A activity in the forebrain, and suppression of microglial activation and proinflammatory cytokine release (Dong and Zhao, 2023).

In addition to their anti-inflammatory properties, active plant compounds like ferulic acid, ginsenoside Rg1, and peptides from soybeans, leaves, and grains (e.g., pEL, rubimetide, and soy-deprestatin) have demonstrated antianxiety and antidepressant effects via gut-brain regulation (Dong and Zhao, 2023; Yang et al., 2022; Mizushige, 2021). These peptides are thought to activate serotonin, dopamine, and GABA systems in the brain through their interaction with the gastrointestinal tract and vagus nerves. Ferulic acid and ginsenoside Rg1 have also been shown to reduce blood corticosterone levels and brain glycogen, as well as alleviate dysbiosis in the colon by modulating microbiome composition and microbial metabolism.

Regarding their effects on impaired neuroplasticity in limbic structures, flavonoids have been found to exhibit antidepressant properties by increasing BDNF and 5-HT levels in these regions (German-Ponciano et al., 2022). Ginsenoside Rg1, ginkgolide A, curcumin, cannabidiol, lavender oil, extracts from Hericium erinaceus mushrooms, and other traditional African medicinal plants have been shown to regulate BDNF/cAMP/CREB signaling in the forebrain (Yang et al., 2022; Gutiérrez-Rodelo et al., 2023; Muller et al., 2021). Lavender oil, in particular, enhances dendritic complexity in primary neurons compared to corticosterone-treated neurons. Interestingly, lavender oil exerts its antidepressant effects without binding to monoamine transporters, neurotransmitter receptors, or GABAergic properties. Its mechanism includes moderate inhibition of voltage-dependent calcium channes (VDCC), which differentiates it from classical antidepressants (Muller et al., 2021).

## 7 Discussion

As discussed above, antidepressive pharmacotherapies are primarily focused on monoaminergic and glutamatergic neurotransmission, which have been shown to influence neuroplasticity in brain limbic structures and mitigate depressive symptoms. However, the clinical utility of currently available antidepressants is limited by insufficient effectiveness and numerous side effects in patients. This highlight the necessity to identify new molecular targets involved in the pathogenesis of depression and to discover compounds with greater therapeutic potential than those offered by current pharmacotherapy.

Animal models provide a unique opportunity to evaluate the effects of potential drugs in the context of a wide range of biological and behavioural actions and play a crucial role in drug development, as well as being used to determine drug efficacy and predict antidepressant efficacy. Rodent models of depression that satisfy critical criteria of face, predictive, and construct validity are most often based on acute, chronic, or social stress paradigms, or genetic predispositions (Duman, 2010; Willner et al., 2019). Traditionally, animal models have been employed to assess drug efficacy and predict antidepressant potential. However, the increasing global burden of TRD requires the development and validation of new preclinical models tailored to this disease.

The Lancet-World Psychiatric Association Commission on Depression (2022) has emphasised that the development and testing of therapies targeting early intervention and novel disease mechanisms is a critical priority. In this regard, variants of the diathesis-stress animal model, also referred to as the vulnerability-stress model, are consistent with these recommendations. The diathesis-stress model posits that an individual predisposed to vulnerability (biological or psychological) may develop depression following exposure to an acute stressor. This vulnerability may encompass biological factors (e.g., genetic predispositions, endocrine imbalances, inflammatory responses, or altered brain connectivity) or psychological traits (e.g., temperament, personality, past experiences, or beliefs) (Monroe and Simons, 1991). These elements are increasingly utilized in animal models with high translational potential to generate novel, rapid-acting antidepressant drug candidates (Papp and Willner, 2023; Papp et al., 2024).

Vulnerability factors stemming from early life stress, social stressors, genetics, or experimental manipulations contribute to the development of depression-like symptoms in animal models. These conditions are associated with alterations in energy metabolism, hypothalamic-pituitary-adrenal (HPA) axis dysfunction, genetic factors, and behavioral disturbances, all of which are relevant to the pathophysiology of depression in humans (Virijevic et al., 2024; Brivio et al., 2022; Willner and Belzung, 2015). While animal models cannot fully replicate the complexity of human depressive disorders, they successfully simulate core symptoms of depression, elucidate underlying mechanisms, and allow the testing of specific hypotheses regarding novel molecular targets. Moreover, animal models are critical for evaluating the potential of newly discovered mechanisms and for assessing the efficacy of drug candidates. Although limitations exist, these models remain invaluable for advancing our understanding of depression and the development of innovative therapies.

In their 2022 review, Borbély et al. highlighted that many phase II clinical trials focus on the effects of drug candidates in treatment-resistant depression (TRD), emphasizing the urgent need for therapies with efficacy in this challenging population. This trend is mirrored in preclinical studies, where antidepressant candidates are often tested in TRD models. Demonstrating efficacy in such models significantly increases the likelihood of success in treating mood disorders. It appears that neuroplasticity-based mechanisms, such as TrkB dimerization and BDNF binding, likely represent a common pathway for many antidepressant therapies, underscoring the essential role of plasticity mechanisms in the long-term efficacy of pharmacotherapy. Understanding and enhancing these mechanisms may be key to improving antidepressant outcomes.

Interestingly, the mechanisms of action of many novel antidepressant drugs have shifted focus to the glutamatergic-GABAergic interplay rather than traditional monoaminergic neurotransmitter systems. New drug candidates include GABA-A potentiators, NMDA receptor antagonists or modulators, and mGlu2/3 receptor modulators. Psilocybin formulations and synthetic psychedelics targeting 5-HT2A receptor agonism are also being explored; however, concerns regarding their safety, including potential risks of addiction and other adverse effects, remain a critical area of debate.

Evidence supports the efficacy of rapid-acting therapies in providing a rapid reduction in depressive symptoms, which could improve patients' quality of life and reduce healthcare costs. However, no glutamatergic modulator tested to date has matched the rapid, robust and sustained antidepressant effects of (R,S)-ketamine and esketamine. In addition, the breadth of therapeutic effects of ketamine, such as its antisuicidal properties, anti-anhedonic effects or broader therapeutic potential, remains unmatched. The investigation of novel compounds for these specific indications remains a high priority.

### 7.1 Challenges and questions for future research

One of the significant challenges in developing new antidepressants is the heterogeneity of mood disorders. Despite significant progress, the pathophysiology of depression remains idiopathic and incompletely understood, complicating efforts to identify universally effective treatments.

Key questions for future research include:

Neuroplasticity and Treatment Response: How can neuroplasticity be effectively enhanced to improve treatment outcomes? What are the most promising molecular targets to stimulate plasticity mechanisms?

Mechanistic Contributions: How do intracellular signaling pathways, network activity, neuroinflammatory and neuroendocrine effects, early-life factors, and the microbiota system contribute to the clinical efficacy of antidepressants?

Combination Therapies: What are the additive or synergistic effects of pharmacotherapy when combined with psychotherapy or other interventions, and how can these combinations be optimized?

Addressing these questions will require an integrated approach, combining insights from neurobiology, pharmacology, genetics, and clinical practice. Continued exploration of novel compounds, combined therapies, and personalized treatment strategies, offers the best path forward to advancing the treatment of depression and TRD.
